# Glioblastoma Stem-*Like* Cells, Metabolic Strategy to Kill a Challenging Target

**DOI:** 10.3389/fonc.2019.00118

**Published:** 2019-03-06

**Authors:** Delphine Garnier, Ophélie Renoult, Marie-Clotilde Alves-Guerra, François Paris, Claire Pecqueur

**Affiliations:** ^1^CRCINA, INSERM CNRS, Université de Nantes, Nantes, France; ^2^INSERM CNRS, Institut Cochin, Paris, France; ^3^Université Paris Descartes, Sorbonne Paris Cité, Paris, France; ^4^Institut de Cancérologie de l'Ouest - René Gauducheau, St Herblain, France; ^5^LabEx IGO “Immunotherapy, Graft, Oncology”, Nantes, France

**Keywords:** Glioblastoma, cancer stem cells, cancer metabolism, tumor microenvironment, cancer heterogeneity, cancer plasticity

## Abstract

Over the years, substantial evidence has definitively confirmed the existence of cancer stem-*like* cells within tumors such as Glioblastoma (GBM). The importance of Glioblastoma stem-*like* cells (GSCs) in tumor progression and relapse clearly highlights that cancer eradication requires killing of GSCs that are intrinsically resistant to conventional therapies as well as eradication of the non-GSCs cells since GSCs emergence relies on a dynamic process. The past decade of research highlights that metabolism is a significant player in tumor progression and actually might orchestrate it. The growing interest in cancer metabolism reprogrammation can lead to innovative approaches exploiting metabolic vulnerabilities of cancer cells. These approaches are challenging since they require overcoming the compensatory and adaptive responses of GSCs. In this review, we will summarize the current knowledge on GSCs with a particular focus on their metabolic complexity. We will also discuss potential approaches targeting GSCs metabolism to potentially improve clinical care.

## Introduction

Glioblastoma (GBM) is the most common primary brain tumor in adults, defined as a grade IV glioma according to the WHO (World Health Organization) classification of central nervous system tumors (1). GBM is characterized by a highly infiltrative nature within the surrounding brain parenchyma and a dismal prognosis despite aggressive treatments. Present GBM standard of care, as defined in the Stupp protocol, includes surgical tumor resection followed by radiotherapy with concomitant and adjuvant chemotherapy with Temozolomide. With this therapy, patient median survival is only 18 months with <5% of patients surviving over 5 years (2). This poor response rate can be explained by the almost inevitable GBM recurrence within a year of initial diagnosis in part due to the limitations of surgical resection given GBM propensity for infiltration, but also to an extensive tumoral heterogeneity resulting in a large range of variabilities in crucial biological responses like cell proliferation, invasion, and sensitivity to conventional treatments. At the genetic level, GBM display a highly mutated genome including loss, amplification, or mutation of EGFR (including expression of the constitutively active form EGFRvIII), PDGFRA, NF1, PTEN, RB1, and p53, resulting in the deregulation of many signaling pathways. Furthermore, epigenetic modifications are also well-characterized in GBM, especially on the O^6^-methylguanine methyltransferase (MGMT) gene, a DNA repair enzyme involved in the fixation of damages induced by alkylating agents such as Temozolomide. Inactivation of the MGMT enzyme, following its promoter hypermethylation, correlates to a better prognosis due to the resultant inability of the MGMT enzyme to remove alkyl groups from DNA (3). This heterogeneity becomes even more complex when therapy comes into the picture with the emergence of drug-resistant clones with highly mutable phenotypes (4–6).

At the cellular level, functional GBM heterogeneity can be explained by the existence of multiple cellular subpopulations of cancer cells. In particular, Glioblastoma stem*-like* cells (GSCs) display stem cell properties of self-renewal and multi-lineage differentiation. These cells generate cellular heterogeneity by establishing a differentiation hierarchy leading to a wide range of distinct cell types present in the tumor. Importantly, extensive studies have implicated these GSCs in GBM recurrence. Recently, an increased focus upon this GSCs subpopulation suggests that their eradication is definitively required in order to successfully treat GBM patients.

Normal stem cells are unique in their ability to self-renew, proliferate, and differentiate in various cell types. They are also characterized by poorly developed mitochondria and a strong glycolytic metabolism. Whereas, the metabolic alterations have been included as a hallmark of cancer cells, contradictory results have been reported for GSCs suggesting a metabolic flexibility. The aim of this review is to summarize and emphasize some of the key aspects of GSCs, with a particular focus on their dynamic emergence and metabolic plasticity. Given the obvious need for improvement of current therapies for GBM, we will also present data on how metabolic targeting might be exploited to eradicate GSCs and hopefully improve clinical outcomes.

## Glioblastoma Stem-*Like* Cells

### Definition and Origin of Cancer Stem-*Like* Cells

The cancer stem*-like* cells (CSCs) concept was originally proposed to reconcile the complex phenotypic heterogeneity of tumors and the fact that only a few cancer cells are actually tumorigenic. CSCs possess the capacity to self-renew, initiate a tumor as well as the potential to differentiate to reconstitute the initial tumor mass, including its heterogeneity (7). An increasing amount of evidence based on preclinical and clinical studies demonstrates the importance of CSCs in tumor progression and relapse suggesting that cancer eradication requires killing of CSCs.

Since the CSCs concept emerged in the 1970's, the origin of these cells is still controversial with opposite models to explain their presence in tumors. The initial and traditional theory is based on a hierarchical and unidirectional model, where CSCs constitute a specific and rare subpopulation of cells that possess the unique capacity to repopulate and reconstitute tumor heterogeneity through symmetric self-renewal of the CSCs pool, and asymmetric divisions to generate differentiated cancer cells (8, 9). In this model, CSCs may have emerged after acquisition of mutations in normal neural stem cells. However, this model has been challenged by subsequent studies highlighting cancer cell plasticity occurring in tumors and giving rise to a new stochastic model based on clonal evolution (10–12). In this model, some tumor cells can progressively accumulate mutations and reacquire a self-renewal potential, forming several CSCs clones (13). Therefore, all the cells forming the tumor bulk have the potential to become CSCs through a dedifferentiation process, already underlining the complexity of their characterization

In conclusion, whereas the non-CSCs constitute the tumor bulk and the CSCs are involved in tumor relapse and metastasis, the hierarchy between CSCs and non-CSCs is definitively bi-directional and highly dynamic, adding further complexity to our understanding of the tumor.

### Phenotypic Plasticity of Glioblastoma Stem-*Like* Cells

In Glioblastoma, GSCs were first identified by Singh et al., as a population of cells capable of initiating tumor growth *in vivo* (8). Like their normal counterparts the neural stem cells, GSCs exhibit self-renewing and multilineage differentiation into neurons, astrocytes, and oligodendrocytes, and even transdifferentiation abilities [review in (14)]. However, in contrast to neural stem cells, GSCs display the ability to initiate a tumor upon transplantation and to recapitulate its initial phenotype and heterogeneity. GSCs are highly resistant to chemotherapy (15, 16) and radiation (17), and have been involved in GBM tumorigenicity. Indeed, GSCs are slow-cycling, have the capacity to limit DNA lesions through strong and efficient DNA damage response, and prevent cytotoxicity through high drug efflux by ABC transporters. Recently, several studies have highlighted that GSCs may also be involved in the infiltrative nature of GBM (18–20). In particular, expression level of Wnt5a defines the infiltrative capacity of GBM cells, including in GSCs. In fact, its overexpression in GSCs confers an exacerbated invasive phenotype while its inhibition reduces their invasive potential both *in vitro* and *in vivo*.

In Glioblastoma, several studies demonstrated the bidirectional plasticity between GSCs and more differentiated GBM cells as a result of environmental factors. First, besides promoting the self-renewal of GSCs, hypoxia through HIF2α promotes a stem-like phenotype in the non-stem population by upregulating several stem cell factors, such as Oct4, Nanog and c-Myc (21). Second, as we described before, chemotherapy as well as radiation consistently increase the GSCs pool over time. In fact, therapeutic doses of Temozolomide trigger a phenotypic shift in the non-GSCs population to a GSCs state (22) while radiation increases tumorigenicity through reacquisition of stemness markers and stem-associated properties of GBM cells, in part via a survivin-dependent pathway (23). Recently, it has been suggested that Sox2, a well-known transcriptional factor involved in stemness maintenance, might be central in tumor cell plasticity by regulating dedifferentiation and acquisition of GSCs properties, through a transcriptional regulation of distinct genes set in differentiated tumor cells and GSCs (24). Hyperactivation of the tyrosine kinase c-Met, involved in the reprogramming of induced pluripotent stem (iPS) cells, also induces GSCs reprogramming via a mechanism requiring Nanog (25). Finally, activation of Epithelial-to-Mesenchymal transition (EMT) enables the conversion of non-CSCs in CSCs through both intracellular and extracellular signaling pathways.

Besides genetic and cellular heterogeneities, The Cancer Genome Atlas (TCGA) has established a molecular classification of GBM using transcriptional profiling data of bulk tumor based on dominant genes expressed in each GBM subtype (26, 27). Three main GBM subtypes can be easily distinguished based on molecular signature, therapy responses and patient survival: the mesenchymal (MES), classical, and proneural (PN), (28). Notably, the MES subtype of GBM is associated with relatively poor prognosis compared with that of the other subtypes and shows resistance to conventional therapy. GSCs also display these molecular signatures with distinct activated signaling pathways, biological phenotypes, and residing niches (29–31). In agreement with the worst outcome, MES GSCs are more resistant to radiation and display more aggressive phenotypes *in vitro* and *in vivo* (31, 32). Recent studies have added a layer of complexity in this molecular classification by demonstrating that molecular subtypes are flexible and vary spatially and temporally within the same tumor. First, a study collecting spatially distinct tumor specimens from the same tumor as well as single-cell RNA-sequencing resolution revealed that a single tumor consists of a heterogeneous mixture of tumor cells from different subtypes, with one subtype usually being highly represented (33, 34). Second, the molecular subtype can evolve with time, microenvironment or stress, in particular toward a MES transdifferentiation from the other subtypes (35), in agreement with a higher frequency of the MES subtype at recurrence (33, 36). Furthermore, radiation of PN GSCs up-regulated mesenchymal-associated markers while down-regulating PN-associated markers. Collectively, these studies underlined the strong unstable nature of GBM, fully in agreement with its previous designation as Glioblastoma Multiform. Since the molecular patterns of GBM can partially explain clinical outcomes and predict responses to treatment, this classification should help understanding the GBM tumorigenesis and progression and provide diagnostic and prognostic information, as well as help the development of new therapeutic approaches. However, several clinical trials based on either targeted therapies for specific mutations or subtypes have been completed with no consistent changes in clinical activity [review in (37)].

Taken altogether, these studies underlie the great diversity of GSCs states suggesting that emergence of GSCs, and CSCs in general, should be consider as a phenotypic shift rather than a true dedifferentiation process.

### Characterization and Isolation of GSCs

As we described previously, CSCs in general constitute a very rare population but one of the most important to target, for their unique ability to reconstitute the initial tumor and their strong resistance to therapy. The ability of all cells forming the tumor bulk to shift into GSCs already underlines the complexity of their characterization. The identification of GSCs is classically based on cell surface markers such as CD133 [review in (38)]. It has been shown that CD133 positive GSCs better survive radiation than CD133 negative cells and were able to give rise to a tumor in a xenograft model. However, some later works showed that CD133 negative cells were also able to initiate tumor growth. Furthermore, while both subtypes exhibit similar GSCs enrichments, PN GSCs clearly exhibit CD133 at their surface in contrast to MES GSCs that do not (39, 40). The sets of markers being used to identify GSCs are being constantly updated and includes Sox2, Olig2, Nestin, CD15/SSEA-1, CD44, integrin alpha6, L1CAM, as well as drug efflux transporters like ATP-binding cassette transporters (ABC) [review in (41)]. However, those markers are also expressed by normal stem cells. Furthermore, purified CD133 positive cells are able to re-establish the initial ratio of CD133 positive and CD133 negative cells (11, 12). This was true not only for CD133 marker but also for 15 commonly used CSCs markers including CD44, ABCB5, or cadherin. Finally, in line with CD133 expression, while PN and MES GSCs are able to self-renew both *in vitro* and *in vivo*, bioinformatic analyses have revealed distinct GSCs phenotypes for these two molecular subtypes into full and restricted stem-like phenotypes, respectively (42). Thus, despite many years of investigation, there is no consensus on an appropriate way to identify these cells. It is nowadays commonly accepted that the identification of GSCs and CSCs in general, requires the combination of several markers and also a functional demonstration of their stem cells features such as self-renewal and their ability to initiate tumor growth.

Because of the difficulty to precisely define GSCs specific markers, the isolation of this rare population of cells becomes very delicate. However, the development of relevant *in vitro* models is essential to better understand GSCs biology, to uncover potential vulnerabilities and to identify novel therapeutic targets. Culture methods, initially developed for neural stem cells, have been adapted to enrich primary GBM cultures in GSCs. Post-surgery GBM specimens are mechanically dissociated and can grow in two different phenotypes, each requiring specific media. They can grow as adherent monolayer cells in presence of serum, which represent differentiated GBM cells, or as free-floating tumorospheres enriched in GSCs when cultured in serum-free medium containing EGF and bFGF (43). Importantly, these two phenotypic states, mutually reversible, differ in proliferation rate, invasion, migration, and chemosensitivity. The validation for GSCs enrichments is based on the combinatorial expression of cell surface markers (CD133, CD44, CD15), intracellular stem cell markers (Nestin, Sox2) and most importantly key stem cells features such as self-renewal and tumor initiation. Self-renewal can be evaluated by limiting dilution assay or colony forming cell assay since one CSC is able to form a tumorosphere due to its self-renewal potential. Based on their high expression of drug efflux transporters, another way to determine GSCs enrichment in GBM cultures is to identify the cells which are able to exclude fluorescent dye, defined as the side population (44). High aldehyde dehydrogenase (ALDH) activity is also one feature of CSCs in general, including GSCs (45). Finally, the most rigorous criteria to establish the presence of GSCs in GBM cultures is to test their ability to initiate a tumor *in vivo*. Importantly, in contrast to commercially available cell lines, these primary GBM cultures enriched in GSCs reproduce the overall behavior of GBM in patients, in particular their highly invasive feature. Thus, while not perfect, these *in vitro* GBM models present a unique opportunity to develop effective CSCs-directed therapies.

### Interactions of GSCs With Their Microenvironment

Like normal stem cells, GSCs rely on a similar permissive tumor microenvironment (TME), also called the niche, to retain their exclusive abilities to self-renew and give rise to more differentiated progenitor cells. This niche includes many different cell types from stromal to immune cells with many reciprocal communications essential for GSCs maintenance, survival and proliferation, as well as TME recruitment, activation, programming, and persistence. Moreover, the CSCs niche also has a protective role by sheltering GSCs from diverse genotoxic insults contributing to their enhanced therapy resistance.

#### Cellular Components of the Niches

Mesenchymal stem cells (MSC) have remarkable tumor tropism that allows them to migrate toward and engraft specifically into the tumor sites, especially cells that have escaped the main tumor mass. MSC are multipotent progenitors that can differentiate into adipocytes, osteocytes, and chondrocytes. They are present in GBM, and more particularly in GSCs niches (46). The role of MSC in tumor progression is still debatable since other studies have shown an opposite effect with MSC inducing the inhibition of GBM tumor growth (47, 48). MSC provide supportive signals to GSCs, as indicated by the inverse correlation between glioma patients survival and the percentage of MSC (49, 50). Besides their homing capacity, they also stimulate proliferation, invasion, and tumorigenesis in GBM, in part via the cytokine IL-6 (51). Aside from a communication through direct cell-cell contact, GSCs can also exchange signals with MSC through Extracellular Vesicles (EV) [review in (52)]. EVs are membrane structures secreted by cells in the extracellular media and can transfer various information (DNA, miRNA, mRNA, proteins, lipids…) after their uptake by recipient cells. These EV contribute to tumor progression, either by reprogramming adjacent cells or by the modification of the supportive tumor microenvironment. Interestingly, the diversity of transcriptomic profiles observed in GBM subtypes is also found at the level of EV, contributing to maintain the GBM heterogeneity through the transfer of oncogenic signals and miRNA (53, 54). Moreover, MES and PN GSCs and their differentiated counterparts secrete different kinds of vesicles, and their uptake by recipient cells such that endothelial cells is also subtype-dependent (55). Thus, the use of EV as blood biomarkers recently received much attention as a fast and non-invasive way to detect and follow GBM tumors, identify their subtypes (55), or even as a marker of resistance acquisition to treatment (4). Indeed the EV content is the perfect reflection of the tumor phenotype as well as its microenvironment, and can easily be collected from a blood sample.

Although the brain has long been considered as immune-privileged due to the blood-brain barrier, recent studies have reported the existence of a direct communication between the CNS and the immune system. In Glioblastoma, the TME has been shown to be particularly immunosuppressive, such that tumor cells can escape from immune surveillance. Notably, GSCs have a lower immunogenicity and a higher suppressive activity compared to non-GSCs GBM cells, through several mechanisms: the inhibition of T cells proliferation (56), the proliferation of regulatory T cells (57) and the recruitment of myeloid-derived suppressor cells (MDSCs), via the secretion of macrophage migration inhibitory factor (MIF) or exosomes (58, 59). Additionally GSCs are involved in the tropism of immunosuppressive cells toward the tumor site including tumor-associated macrophages, through the secretion of numerous cytokines or growth factors, such as TGF-β1, SDF-1, or VEGF [review in (60)]. Interestingly GSCs seem to be more sensitive to Natural Killer (NK) cell toxicity, compared to their differentiated counterpart (61, 62). However, to prevent their recognition and elimination by cytotoxic NK cells, some GSCs, such as IDH-mutant GSCs, exhibit significantly lower NKG2D expression to prevent their recognition by NK cells (63). Inversely different soluble factors secreted by immune cells also play essential roles for the biology of GSCs, for example TGFβ secreted by myeloid cells is an inductor of EMT involved in GSCs emergence.

#### Oxygen Is a Critical Component of GSCs Niches

Oxygen concentration plays a fundamental role in stemness maintenance defining several GSCs niches, in particular the perivascular and the hypoxic niches (64, 65). The most frequently described GSCs niche is the perivascular one in which the vascular component plays a crucial role in stemness maintenance and survival as well as GSCs dissemination (66). Accordingly, orthotopic co-implantation of GSCs with endothelial cells increases the GSCs fraction in xenograft tumors. Besides the direct contact between GSCs and endothelial cells, soluble factors produced by these cells also enhance stemness markers (66, 67). Notch signaling, in part through NO, plays a critical role in both GSCs maintenance (68) and GSCs radioresistance (69). In return, GSCs secrete several cytokines or growth factors, such as SDF-1 or VEGF, to promote angiogenesis through endothelial cells proliferation and recruitment (70, 71). Upon particular stimuli, GSCs can transdifferentiate into endothelial cells or pericytes to directly contribute to the perivascular niche (14). Importantly, this transdifferentiation can also transfer to endothelial cells the capacity of resistance and genetic mutations (72). The communication between the perivascular niche and the GSCs is therefore a two-way exchange with benefits to both blood vessels and GSCs.

One hallmark of GBM tumors is the existence of hypoxic zones which have been shown to be enriched in GSCs (73). Indeed, hypoxia directly supports GSCs self-renewal as well as controls stem cell plasticity and non-GSCs reprogrammation through transcriptional regulation via activation of HIF1α, HIF2α, and the Notch pathways, epigenetic regulations and metabolic reprogramming [review in (21, 74)]. HIF2α also activates c-Myc, another key stem cell regulator. Hypoxia also causes the secretion by GSCs of several soluble factors such as TGF-β, an activator of EMT that favors the dedifferentiation of tumor cells, or VEGF and SDF-1 to promote angiogenesis by recruiting mesenchymal stem cells and myeloid cells (71). Other secreted factors induced by hypoxia also include CXCR4, the glucose transporter 1 (GLUT1), Serpin B9, Oct4, lysyl oxidase (LOX), hypoxia inducible gene 2 (HIG2), all these factors being involved in the maintenance and proliferation of GSCs [(74, 75)].

These findings highlight the critical role of the TME in regulating the differentiation status of tumor cells and the plasticity of GSCs and non-GSCs hierarchy. Importantly, each subtype is associated with a particular environment, the MES GSCs being located in hypoxic niches while the PN GSCs are in perivascular niches.

## Metabolic Phenotypes of GSCs

Dynamic changes also occur at the bioenergetic machinery level which strongly contributes to tumor heterogeneity. Indeed, the ability of the tumor mass to face the large need for both biomass precursors and ATP production required by intense tumor proliferation, mainly relies on the metabolic plasticity of cancer cells.

### Altered Metabolism Is a Hallmark of All Cancer Cells

**Figure 1:**
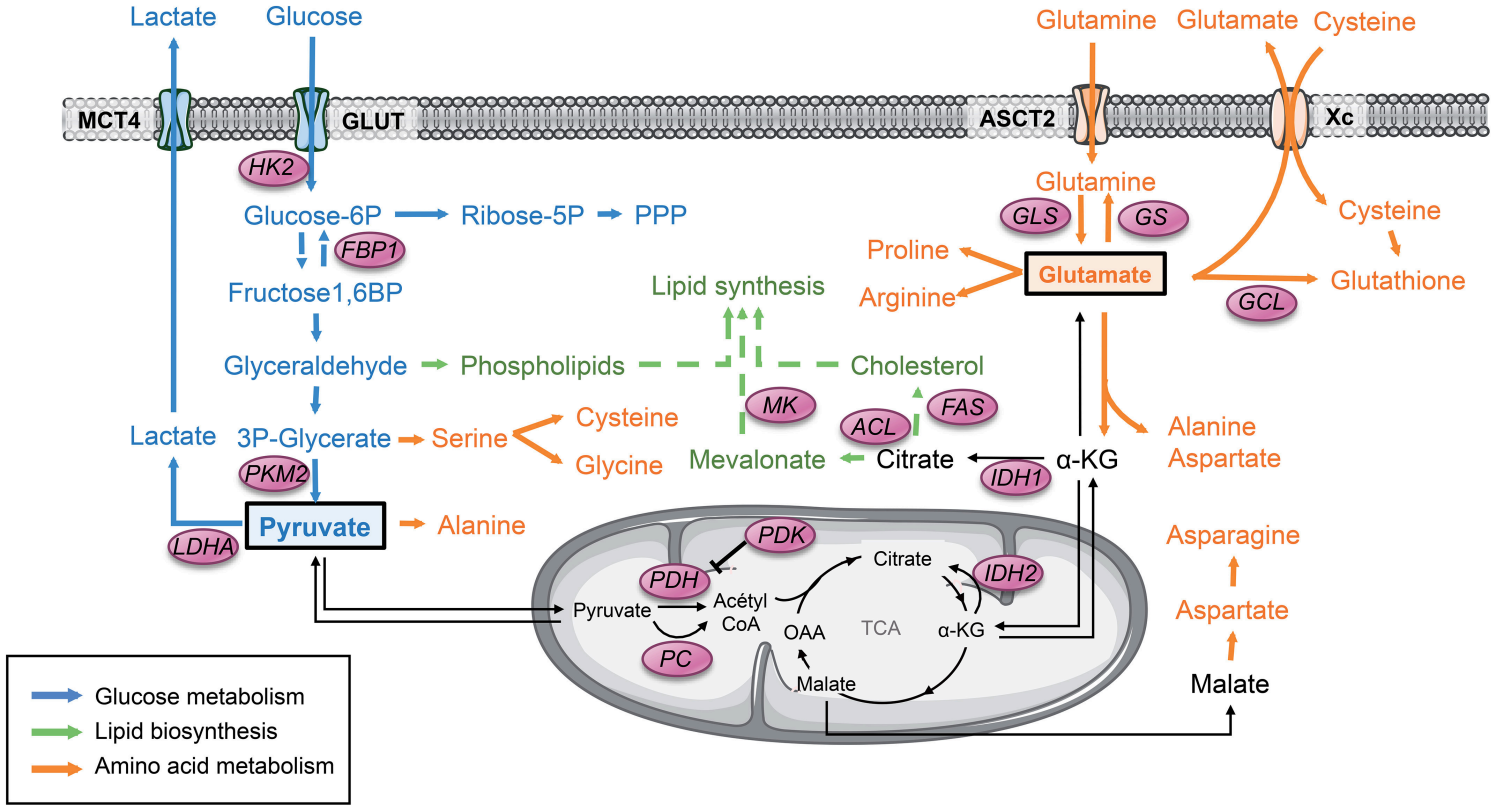
Pyruvate and glutamate are 2 major metabolic hubs in GSCs. Tumor cells usually display a strong glycolytic metabolism. Glucose is uptaken by glucose transporter GLUT and then converted to pyruvate through several enzymes. All along this pathway, the glycolytic products are diverted from this main metabolic road to fuel other biosynthetic pathways such as the PPP as well as lipids and amino acids biosynthesis. PKM2 plays a key role in this dynamic process through conformational modulation. Glycolytic pyruvate will then either be converted to lactate or fuel mitochondrial OXPHOS and the TCA cycle. Another key metabolite that can fuel the TCA is glutamate once converted to αKG. Glutamate is produced either by GLS from glutamine or from glucose. Glutamate is involved in several biosynthetic pathways including amino acids and lipids biosynthesis as well as mitochondrial anaplerosis. Glutamate is also involved in glutathione synthesis, directly and indirectly by providing cysteine to the cells. α-KG, α-ketoglutarate; FAS, Fatty acid synthase; FBP1, Fructose-1,6 bisphosphatase 1; GCL, glutamate-cysteine ligase; GLS, Glutaminase; GS, Glutamine synthetase; GLUT, Glucose transporter 1; HK2, Hexokinase 2; IDH, Isocitrate dehydrogenase; LDHA, Lactate dehydrogenase A; MCT, Monocarboxylase transporter; MK, Mevalonate kinase; OAA, Oxaloacetate; PC, Pyruvate carboxylase; PDH, Pyruvate dehydrogenase; PDK, Pyruvate dehydrogenase Kinase; PKM2, Pyruvate kinase M2; PPP, Pentose phosphate pathway; TCA, Tricarboxylic acid cycle.

In normal cells, glucose homeostasis is reciprocally controlled by catalytic glycolysis/oxidative phosphorylation and the anabolic neogluconeogenesis pathway. In the catabolic reaction, glucose is converted to pyruvate which can be further metabolized to mitochondrial acetylCoA to fuel the Tricarboxylic acid cycle (TCA) and oxidative phosphorylation (OXPHOS), or to cytosolic lactate. Importantly, whereas OXPHOS is bioenergetically highly efficient with 36 molecules of ATP produced from one molecule of glucose, the direct conversion of glucose to lactate, usually occurring in absence of oxygen, produces only 2 molecules of ATP. Paradoxally, in 1924, Otto Warburg discovered that tumor cells use large amounts of glucose to produce lactate, even in the presence of oxygen (Figure 1). This counter intuitive energy generation pathway occurs in most cancer cells, independently of mitochondrial functional integrity. In fact, by increasing their glycolytic and anaplerotic fluxes, tumor cells cope with increased bioenergetic as well as biosynthetic needs. This finding resulted in the development of 2-[18F]-fluoro-2-deoxy-D-glucose positron emission tomography (PET) to detect glucose uptake and lactate production for tumor imaging. In 2011, altered energy metabolism has been integrated as a fundamental core hallmark of cancer cells (76).

Traditionally, metabolic reprogramming is viewed as driven by oncogenic gain-of-function events or loss of tumor-suppressors. For example, tumor suppressor gene p53, mutated in more than 50% of human cancers, including GBM, triggers glycolysis besides its key roles in genetic instability, tumor progression, and metastasis. Loss of PTEN leads to the constitutive activation of AKT1, which stimulates glucose uptake by enhancing GLUT4 expression and activating HK2 (77, 78). Finally, activation of c-MYC also induces glycolysis by inducing LDH-A and PDK1 expression facilitating the production of lactate (79). Interestingly, in the past 2 decades, it was shown that loss-of-function mutations of the TCA cycle enzymes succinate dehydrogenase and fumarate hydratase were implicated in the pathogenesis of paragangliomas, pheochromocytomas, and renal cell cancers (80). Notably, mutations in the genes encoding isocitrate dehydrogenase (IDH)-1 and IDH-2 have been described in 5% of *de novo* GBM and more than 90% of secondary GBM [review in (81)]. In fact, since 2016, the CNS WHO divided GBM in 2 main groups (1) IDH-wildtype (about 90 % of cases), which corresponds most frequently to *de novo* GBM and predominates in patients over 55 years of age, and (2) IDH-mutant (about 10 % of cases), corresponding to secondary Glioblastoma arising from lower grade diffuse glioma and preferentially arises in younger patients (1). Of note, in GBM, IDH mutants are consistently classified as PN. Tumor-derived IDH mutations disrupt their normal catalytic activity that converts isocitrate to α-ketoglutarate (α-KG) to a remarkable neomorphic activity that converts α-KG to D-2-hydroxyglutarate (D-2-HG), now referred to as an oncometabolite (82). Besides being involved in multiple metabolic pathways, α-KG is also a co-factor for several α-KG-dependent dioxygenases including prolylhydroxylases (PHD) involved in HIF stabilization, histone demethylases and the TET family dioxygenases involved in epigenetic modifications. Interestingly, D-2-HG that is structurally highly similar to α-KG, acts as a competitive inhibitor leading to dioxygenases inhibition and singular methylation profile.

Importantly, these mutations in genes encoding for important metabolic enzymes raised the possibility that under certain conditions, altered metabolism could be the cause and not the consequence of cancer transformation. In line with this challenging notion, several studies have evidenced a retrograde mitochondrial-to-nucleus signaling. For example, disruption of mitochondrial integrity generates singular nuclear genes expression profiles, including genes involved in metabolism (83). Thus, tumor cells might exhibit increased metabolic autonomy in maintaining an anabolic phenotype with oncogene and tumor-suppressor gene originated through evolution as components of metabolic regulation rather than cancer-driving mutations driving metabolic pathways (84). This notion would explain why cancer cells with different genetic alterations display similar metabolic phenotype whereas cancer cells with identical genetic alterations have different metabolism.

### Metabolic Plasticity of GSCs

In contrast to the proliferating tumor mass, GSCs are slowly proliferating and reside in specific niches requiring constant metabolic adjustments in order to adapt to transient bioenergetic crisis caused by hypoxia or nutrients deprivation. While they also display metabolic alterations, the metabolism of GSCs have been shown to deeply influence their maintenance and survival.

#### GSCs Rely on Both Oxidative and Non-oxidative Glucose Metabolism

GSCs, as most CSCs, have been described as relying mostly on glycolysis. Glucose is uptaken by the cells through the glucose transporters (GLUT), in particular GLUT1 and GLUT3, and converted to pyruvate through several enzymatic reactions (Figure 1). Pyruvate represents a critical metabolic control point, as it can be converted to lactate by LDH-A or imported within the mitochondria to be converted to acetyl coenzyme A (AcetylCoA) by pyruvate dehydrogenase (PDH) to fuel the TCA cycle. Activation of the transcription of both Pyruvate dehydrogenase kinase (PDK), which phosphorylates and inactivates the catalytic domain of PDH, and the Lactate dehydrogenase A (LDH-A), in particular through HIF-1, has been reported in GSCs. As a result, pyruvate is actively shunted away from the mitochondria and converted to lactate by LDH-A, corresponding to a non-oxidative glucose metabolism. However, Marin-Valencia et al. have elegantly demonstrated using 13C-nutrient labeling in orthotopic murine models that GSCs are not confined to non-oxidative glycolysis (85). Indeed, they show that pyruvate is converted to lactate but also channeled through PDH with a significant contribution of glucose carbons to the TCA cycle. Collectively, these results demonstrate that GSCs use glucose to produce energy and biosynthetic precursors through both non-oxidative and mitochondrial oxidative pathways. Since these tracers are not radioactive, this approach is feasible with GBM patients when tumor resection is planned in the course of the treatment. Importantly, similar metabolic profiles of both oxidative and non-oxidative glucose fates were observed in extracts of surgically-resected tumors obtained from GBM patients infused with 13C-Glucose (86).

Furthermore, depending on oxygen availability, a reciprocal metabolic switch has been reported between glycolysis and the pentose phosphate pathway (PPP), an alternative anabolic pathway to glycolysis, which produces ribose-5-phosphate and NADPH for nucleic acids and fatty acids synthesis in GSCs. Indeed, anabolic PPP is highly active in rapidly proliferating tumor cells but suppressed under hypoxia, switching to glycolysis (87). The isoform M2 of pyruvate kinase (PKM2) plays a critical role in this metabolic switch. In contrast to PKM1, which exists in a constitutively tetrameric active form, PKM2 exists under a dimeric inactive form and a tetrameric active form. The dimeric PKM2 results in the accumulation of upstream glycolytic intermediates, in particular fructose-1,6-phosphate (FBP), favoring their redistribution toward other biosynthetic pathways through the PPP. However, accumulation of FBP induces the association of dimeric forms into tetramers, which in turn leads to lactate production, until the level of FBP is reduced leading to the tetramer dissociation into dimers. Thus, the dynamic dimer:tetramer ratio of PKM2 determines whether carbons from glucose are converted into lactate via pyruvate or channeled into building block synthesis.

#### Mitochondrial Function Is Critical for GSCs Survival

While the above reported studies show that GSCs mainly rely on glycolysis, several other studies showed that these cells possess a preference for mitochondrial oxidative metabolism. In many tumor types including Glioblastoma, growing evidence has demonstrated that quiescent or slow-cycling CSCs are less glycolytic, consume less glucose, and produce less lactate, whereas they contain higher ATP levels than their differentiated cancer counterparts (88). Coupling between EMT and enhanced mitochondrial respiration has also been reported. Moreover, some CSCs have an increased mitochondrial mass and enhanced oxygen consumption rates (89, 90). One striking difference with non-GSCs cancer cells is that GSCs lack mitochondrial reserve capacity suggesting that they fully oxidize substrates pool in contrast to differentiated cells exhibiting significant bioenergetic reserves.

In either case, mitochondrial function is critical and plays a crucial role in CSCs functions including stemness and drug resistance. Inhibition of PGC1α, the master regulator of mitochondrial biogenesis, reduces stemness properties of breast CSCs (91) while NANOG, a pluripotency gene, induces tumorigenesis through metabolic reprogramming to OXPHOS and fatty acid metabolism (92). Increased OXPHOS as well as PGC1α expression seems to be related to chemoresistance in CSCs (93–95). In fact, mitochondrial mass tracking using fluorescent probes has been described as a simple and efficient tool to identify CSCs (96). Thus, mitochondrial metabolism is critical for GSCs maintenance.

#### Glutamine Metabolism in GSCs

**Figure 2:**
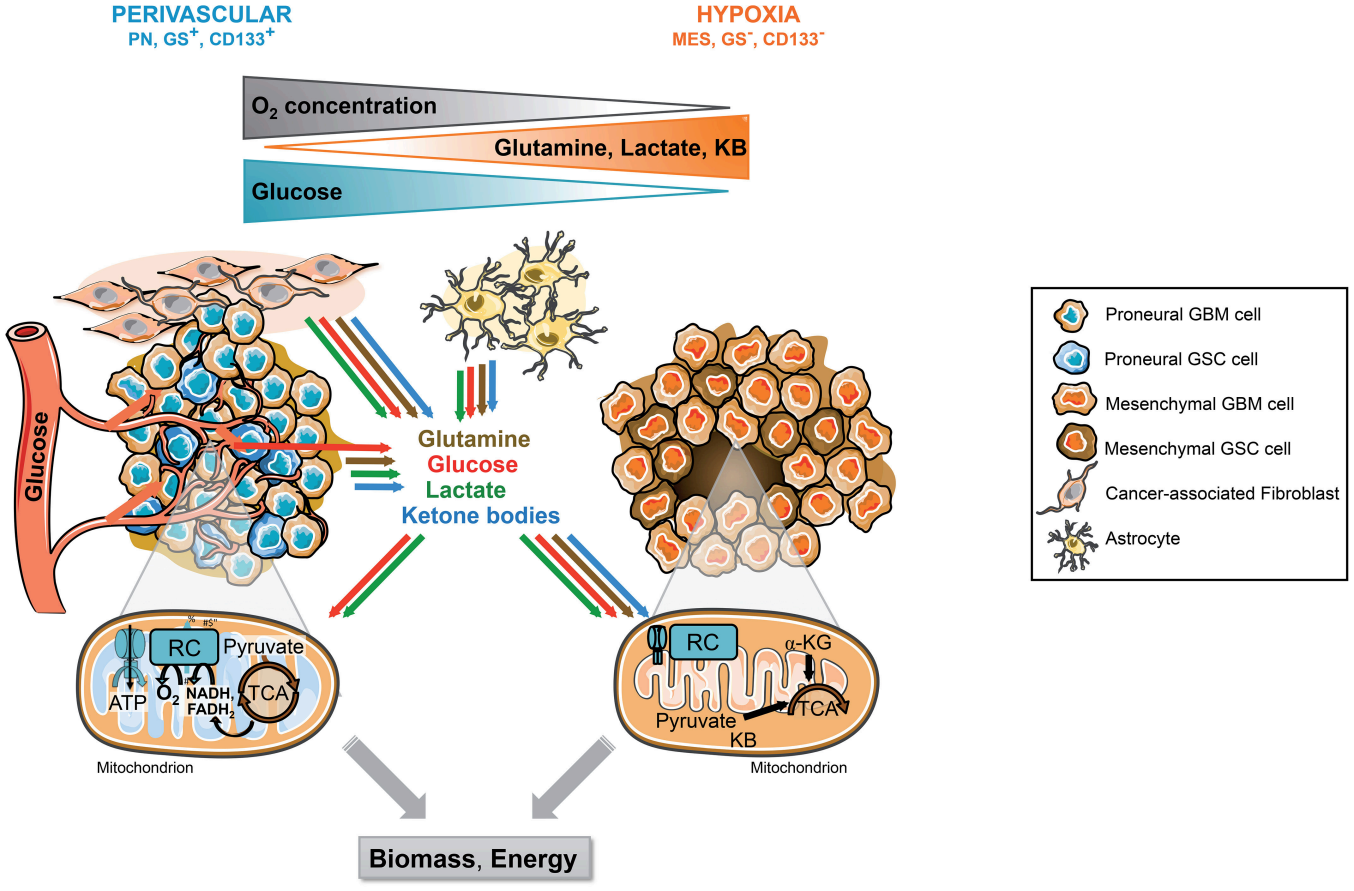
Tumor microenvironment and molecular signature, 2 drivers of GSCs metabolic phenotype. GSCs reside in singular tumor microenvironment in GBM called the perivascular and the hypoxic niche. Each niche is associated with specific molecular signature and metabolic phenotype. The perivascular GSCs display a PN signature and a strong glycolytic metabolism mainly based on blood glucose. In these cells, glucose can be directly converted to lactate or oxidized within the mitochondria through OXPHOS. Furthermore, these cells highly express GS allowing the direct synthesis and secretion of glutamine within the TME. In contrast, hypoxic GSCs usually belong to the mesenchymal subtype and do not express GS. These cells are residing in a harsher environment display flexible metabolism fueled by lactate, ketone bodies, amino acids, including glutamine, released in the microenvironment by other cancer cells and/or stromal cells including astrocytes or cancer-associated fibroblasts. In absence of O2, these substrates supply a truncated TCA cycle allowing the generation of energy and biosynthetic precursors such as lipids or nucleic acids, as well as antioxidant glutathione synthesis. CAF, Cancer-associated fibroblast; GBM, Glioblastoma; GSCs, Glioblastoma *stem-like* cells, GS, Glutamine synthetase; αKG, Alpha-ketoglutarate; KB, Ketone bodies; MES, Mesenchymal; OXPHOS, Mitochondrial oxidative phosphorylation; PN, Proneural; TCA, Tricarboxylic acid cycle.

In addition to glucose, amino acids can also be channeled into the mitochondrial TCA, as their catabolism results in the production of TCA intermediates. Glutamine has an important role in cell growth and energy metabolism, and glutamine addiction has been proposed as a mark of GBM (97). Following the entry of glutamine into the cell via its transporter ASCT2 (or SLC1A5), the first step of glutamine catabolism is its conversion into glutamate through glutaminase (GLS) (Figure 1). Glutamate is a major metabolic hub: it can replenish the cells in lipid biosynthesis precursors and TCA intermediates through mitochondrial anaplerosis, generate *de novo* reduced glutathione, a major anti-oxidant molecule (98) either directly through its combination with cysteine by glutamate-cysteine ligase (GCL) or indirectly allowing cysteine import through cysteine/glutamate transporter Xc (or SLC7A11), or involved in the synthesis of non-essential amino acids, purine, and pyrimidine through aminotransferases (99). In human hematopoietic stem cells, glutamine availability modulates their differentiation either toward the erythroid or the myelomonocytic lineage (100). In GSCs, glutamine deprivation reduces cell proliferation, in particular by reducing mitochondrial anaplerosis and energy production (39). However, glutamine addiction has not been observed in all GSCs (39, 101). Indeed, GLS activity and glutamine anaplerosis is dispensable in GSCs expressing Glutamine synthetase (GS), which controls glutamine homeostasis by catalyzing the opposite reaction of GLS and is highly expressed in GSCs as compared to tumor cells. Based on these studies, we proposed a model where in GS-positive cells, glucose is used to synthetize glutamate that is subsequently converted into glutamine through GS to sustain nucleotides biosynthesis. In contrast, in GS-negative cells, or when GS was inhibited pharmacologically or molecularly, glutamine deprivation decreased cell proliferation as well as self-renewal mostly through reduced nucleotides biosynthesis (Figure 2). Importantly, glutamine-dependency is not observed in non-GSCs and is restricted to one particular GSCs subtype, the MES one.

#### Lipid Metabolism of GSCs

Lipid metabolism is another source of metabolic intermediates and energy for processes involved in cell transformation and tumor progression. Cancer cells can fulfill their avidity for lipids either by increasing exogenous lipid uptake or endogenous production through *de novo* synthesis (Figure 1). A major outcome of both glucose and glutamine metabolism is the production of citrate to produce biomass needs required for cell proliferation. After its import into mitochondria, glycolytic pyruvate is decarboxylated into acetylCoA, which will be condensed with the glutamine-derived TCA intermediate oxaloacetate (OAA) to generate citrate. Citrate can then be exported out of the mitochondria and cleaved by ATP citrate lyase (ACL) to regenerate OAA and acetylCoA. Interestingly, in absence of glutamine, some tumor cells generate both acetylCoA and OAA directly from glucose through the pyruvate carboxylase (PC) pathway (102). Glutamine can also contribute to acetyl-coA production in the absence of glucose. Usually, glutamine-derived glutamate is converted to the TCA cycle intermediate αKG mainly through transamination, which transfers nitrogen from glutamate to alanine or aspartate, since Glutamate Dehydrogenase (GDH) activity is usually inhibited in cancer cells (98). In absence of glucose, cells engage an alternative pathway of αKG production either through enhanced activity of GDH (103) or reductive carboxylation through reverse activity of isocitrate dehydrogenase (IDH) to generate isocitrate and subsequently citrate (104). This latter pathway has been demonstrated when mitochondria are defective (105) or under hypoxia (106, 107). Importantly, glutamine-reductive carboxylation shown to be of particular importance for sustaining cell proliferation has also been observed in GBM cells, including GSCs [(98), Oizel unpublished data].

Besides lipid anabolism, lipid catabolism seems critical for CSCs self-renewal. Indeed, some studies have shown that cancer stem cells can also rely on active fatty acid oxidation for their maintenance and function (108). For example, inhibition of fatty acid oxidation by etomoxir decreases viability and tumorosphere formation in breast cancer stem cells while it has no effect on non-stem cancer cells (109).

Finally, the mevalonate pathway is an essential metabolic pathway allowing the production of 5-carbon building blocks providing cells with bioactive molecules, in particular cholesterol and coenzyme Q as well as molecules involved in signal transduction, which have been shown to be crucial for different cellular processes, including cell proliferation, differentiation, and survival [review in (110)]. Upregulation of several enzymes upstream of the mevalonate pathway has been associated with CSCs enrichment at least in breast tumor cells (111).

In conclusion, GSCs dispose of numerous bioenergetic and biosynthetic redundant pathways in order to fulfill their requirement to sustain their survival, growth, and invasion. All GSCs are able to sustain the emergence of a primary tumor *in vivo*, independently of their predominant glycolytic or oxidative metabolism. Their metabolic flexibility allows their adaptation to harsh microenvironments, where nutrients or oxygen can be scarce. Importantly, in contrast to non-GSC, their metabolic dependency to OXPHOS sets mitochondrial metabolism as potential therapeutic target to efficiently eradicate GSCs.

### Drivers of GSCs Metabolic Phenotypes

#### Metabolic Phenotypes of GSCs Mirror Molecular Signature

Several studies have characterized the metabolic phenotype of the different subtypes of GSCs, either based on stemness expression markers such as CD133 expression or on the molecular signature (39, 112–117). Interestingly, all studies converge toward the proneural subtype, also characterized as CD133 positive or full stem-like phenotype, displaying a strong glycolytic metabolism (Figure 2). In contrast, the mesenchymal subtype, CD133 negative or restricted stem-like phenotype, displays higher metabolic flexibility with both glycolytic and oxidative metabolisms. In particular, as described previously, glutamine dependency relies on GS activity, which is only observed in MES GSCs. Furthermore, in contrast to PN GSCs, MES GSCs easily bypass targeted metabolic inhibition through a wide range of metabolic adaptation. Importantly, these findings highlight that first, different GSCs can have similar metabolic profile despite being derived from independant tumors with different driver mutations. Conversely GSCs derived from identical tumor can exhibit different metabolic features depending on tumor specimen localization or molecular signature. Second, the molecular signature correlates with metabolic flexibility and the spatial distribution of various TME, namely hypoxic and perivascular niches. For example, in agreement with they dominant glycolytic phenotype, the proneural subtype localized in tumor zones surrounded by functional vasculature will easily have access to oxygen and blood glucose. In contrast, the mesenchymal GSCs localized in hypoxic zone display higher metabolic flexibility allowing them to use a wide range of nutrients in order to sustain their survival and proliferation in a relatively poor microenvironment. Importantly, these various metabolic features of GSCs residing in different tumor areas allow the establishment of a metabolic cooperation between tumor cells.

#### Tumor Microenvironment Mitigates Metabolism of GSCs

GSCs are able to adapt their metabolism by displaying various metabolic and bioenergetic abilities depending on energy-rich nutrients or energy-producing mitochondrial metabolites. This is usually dictated by oxygen and nutrients supply from the TME either through the tumor vasculature or through metabolic cooperation between cells which is often not uniformly distributed across the tumor bulk. Several studies have shown the impact of the TME on CSCs metabolism, in particular in GBM. First, as we previously described, one remarkable characteristic of GBM microenvironment is hypoxia due to aberrant tumor vasculature. Low oxygen conditions are mainly mediated by the transcription factors HIF, which regulate the expression of a large panel of genes involved in several key biological functions, including cell metabolism (118). In particular, HIF-1α activates the transcription of genes all along the glycolytic pathway, from the glucose transporters GLUT1 and GLUT3 to key glycolytic enzymes such as hexokinases (HK), PKM2, or LDH-A. Besides directly activating aerobic glycolysis, hypoxia also triggers the epithelial-mesenchymal transition (EMT), a characteristic of embryonic development playing critical roles during organogenesis. Accumulating evidences indicate that EMT is of paramount importance in a plethora of cancer-related events, including acquisition of both stem cell-like properties and mesenchymal characteristics (74). During tumor progression, EMT has been shown to increase the ability of cancer cells to invade and dissiminate, as described previously, to favor CSCs emergence. Among the transcription factors involved in EMT phenotype, Snail has been identified as a key repressor of Fructose1,6 biphosphatase (FBP1) expression in breast cancer stem cells (119). In this study, Snail silences FBP1 expression through methylation of its promoter, providing metabolic advantages to breast CSCs. Indeed, loss of FBP1 induces glycolysis, increased glucose uptake, macromolecules biosynthesis and a constitutively active form of PKM2.

Metabolic reprogramming can be accelerated by TME acidification (120). In fact, lactate is not only a metabolic waste for glycolytic cells but is also a metabolic fuel for oxidative cells (121). Indeed, in 2008 Sonveaux et al. suggested a symbiotic relationship among cancer cells in the TME where cancer cells distal to a blood vessel would be deprived of oxygen in contrast to cancer cells close to the blood vessel (Figure 2) (122). In this model, perivascular cells would spare glucose for hypoxic cells. Then, the distal hypoxic cells would convert glucose to lactate, which could then be imported into perivascular cells and converted to pyruvate for mitochondrial oxidation. This metabolic interplay between glycolytic and oxidative cells, called the “reverse Warburg effect,” also occurs with the stromal compartment that surrounds the tumor. Indeed, the Lisanti group showed that stromal cells, in particular cancer-associated fibroblasts (CAFs), increase their aerobic glycolysis resulting from enhanced mitochondrial turnover and generate excessive lactate, pyruvate, and other ketones bodies, which are secreted into the intracellular space (123). These metabolites are then re-used by cancer cells for OXPHOS. Thus, the stromal cells feed the cancer cells with lactate and other metabolites and this metabolic symbiosis limits TME acidification. The utilization by cancer cells of the high-energy stromal metabolites pyruvate, lactate, and ketones may increase the transcriptional expression of gene profiles normally associated with stemness, including genes commonly upregulated in embryonic stem cells (121). Interestingly, hypoxic tumor cells through mitochondrial reductive carboxylation, independently of oxygen availability, can also use these metabolites. In the brain, glycolytic oligodendrocytes and astrocytes also export lactate through MCT1 and MCT4 (124). Furthermore, astrocytes, which have been shown to highly express GS, generate and secrete glutamine, which can then be used by GS negative tumor cells to support their growth (101).

Finally, CSCs communicate with the surrounding microenvironment by direct cell-cell interaction through gap junctions to execute coordinated programs required for growth, differentiation, and therapeutic response. In the past decade, several publications have reported an important role of tunneling nanotubes (TNT) in direct intercellular communication in GBM. TNT are long cytoplasmic bridges that enable long-range and direct communication, including metabolites, mitochondria and vesicles transfer, between connected cells (125). Mitochondria are the TNT cargos most widely studied so far. TNT-mediated mitochondria transfer was reported in GBM but also in many different cancer types, both between cancer cells, and between cancer cells and normal cells of the microenvironment such as MSC (126). A direct effect of the TNT-mediated transfer of mitochondria is the modification of the target cell energetic metabolism, with increased OXPHOS and ATP production (126–128). This has functional consequences for cancer cells as it enhances their proliferative and migratory properties as well as their capacity to develop resistance to therapeutic treatment.

## Metabolic Targeting of GSCs

GSC involved in GBM poor response to treatment and relapse represent the tumor cornerstone. With the identification of key metabolic features involved in their survival, growth, invading properties and self-renewal, one possible therapeutic strategy would be targeting the biochemical energetic reactions occurring in GSCs.

### Inhibition of IDH-Mutant

Since IDH mutations are present in only 5% of *de novo* GBM while its occurrence increases to 95% in secondary GBM (129), targeting IDH-induced metabolic alteration might open new therapeutic avenues, at least in secondary GBM. As described previously, IDH mutations result in metabolic alteration, including the production of the oncometabolite D2-HG, epigenetic dysregulation via inhibition of αKG-dependent histone and DNA demethylases, and differentiation blockade. Several compounds inhibiting IDH-mutants have been developed and tested in anti-tumor therapy [see review in (130)]. The majority of these studies have been realized in another tumor type, the acute myeloid leukemia (AML), which has been shown to frequently harbor IDH-mutant. Several mouse models have been engineered to express IDH-mutant specifically in hematopoietic tissue leading to hematologic malignancy (131). Genetic knockdown or pharmacologic inhibition of IDH-mutant in these models led to a decrease in D2-HG production and tumor cell growth while inducing cellular differentiation. These encouraging preclinical results provided a proof-of-concept for a targeted treatment of IDH mutants in AML and clinical trials are underway for AML patients (130).

These compounds are now tested in GBM. However, they may not be as effective in all IDH-mutant tumors. Indeed, inhibitors of IDH-mutants delay growth and promote differentiation in some IDH-mutated GBM cells but not all (132, 133). Furthermore, despite a robust ability to lower D2-HG in GBM cells and tumors, they are unable to reverse epigenetic deregulation. Nevertheless, the presence of IDH-mutant and/or high levels of D2-HG might introduce therapeutic vulnerability to different agents. For example, tumors harboring an IDH mutation display increased sensitivity to inhibition of oxidative mitochondrial metabolism (134), depletion of coenzyme NAD (133) and chemotherapy (135). Importantly, other studies have shown both *in vitro* and in patients that inhibiting mutant IDH could confer radiation resistance to some therapies or can antagonize the effects of radiation therapy in glioma (136, 137). Thus, whereas encouraging results have been observed for AML patients, IDH-mutant targeting in GBM definitively requires more investigations. In particular, long-term studies are definitively required to determine the impact of these compounds on tumor relapse in order to define whether these compounds also target cancer stem-*like* cells.

### Metabolic Targeting Requires Multiple Bioenergetic Pathway Inhibitions

**Figure 3:**
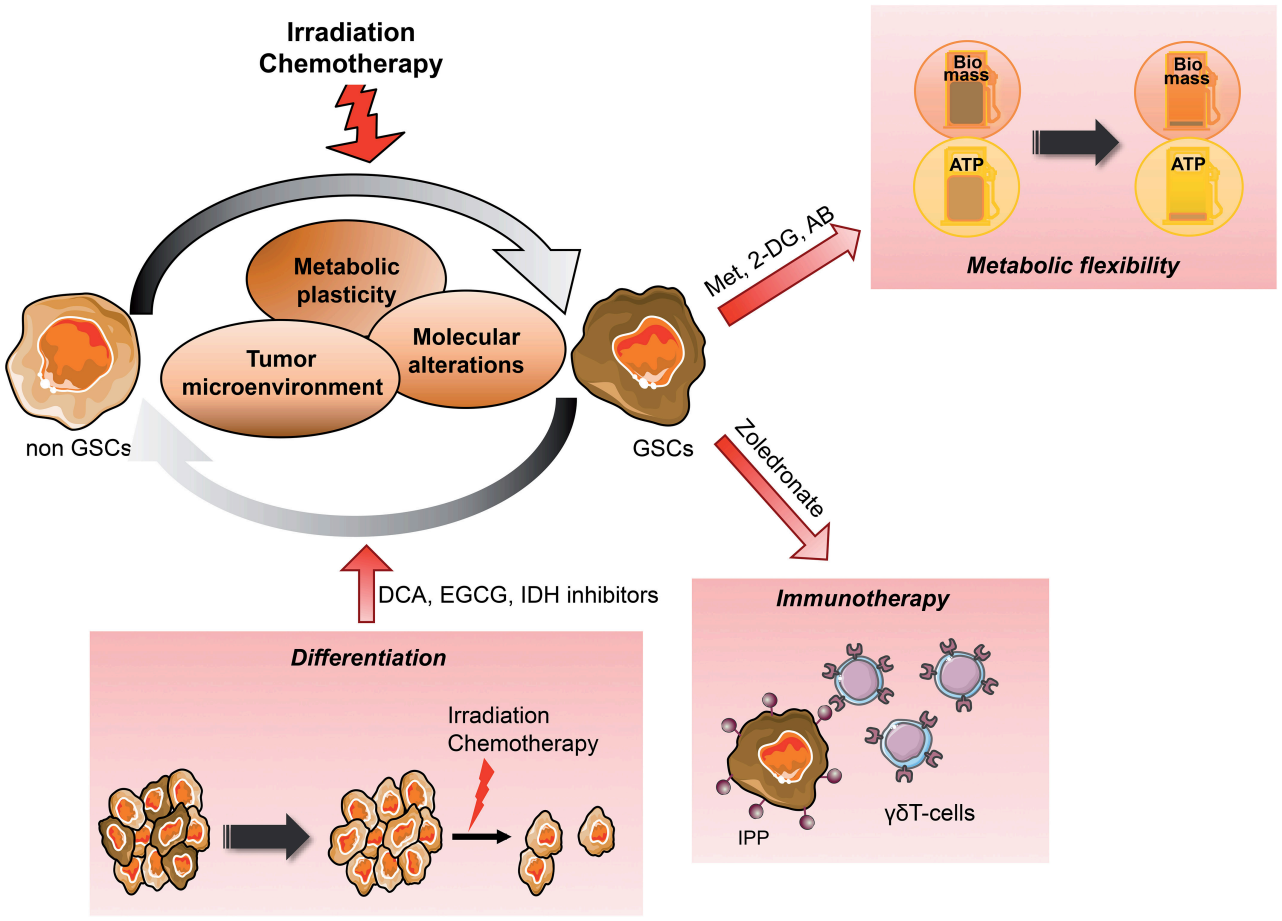
Metabolic targeting of the dynamic emergence of GSCs from differentiated GBM GSCs emerge from differentiated tumor cells, which can reacquire stem cells properties such as self-renewal, multilineage differentiation and the ability to give rise to the initial heterogenous tumor *in vivo*. This dynamic differentiation/dedifferentiation balance is driven by the TME, molecular events as well as their metabolic phenotype. Thus, metabolic targeted therapy appears as a potential novel therapeutic avenue, in particular when combined to conventional treatment such as radiation therapy. Several angles can be taken to eradicate GSCs from the tumor, reducing their metabolic plasticity and/or metabolic cooperation with the TME, inducing their differentiation to sensitize them to treatment or increasing their recognition by immune effectors. AB, Antibiotics; CAF, Cancer-associated fibroblast; DCA, Dichloroacetate; 2-DG, 2-Deoxyglucose; EGCG, Epigallocatechine gallate; GSCs, Glioblastoma *Stem-like* cells; IPP, Isopentenyl pyrophosphate; Met, Metformin; MSC, Mesenchymal stem cell.

CSCs display highly plastic metabolic profile allowing them to fulfill bioenergetic requirements and this flexibility seems to be required for their survival (138). Since CSCs have been shown to be enriched in mitochondrial mass and relying heavily on OXPHOS, disrupting this pathway has become attractive as a therapeutic strategy (Figure 3). Accordingly, different classes of mitochondrial inhibitors have been reported to decrease stemness and invasion properties as well as increasing cell death. For example, a large number of retrospective clinical studies have revealed that Metformin, a first-line diabetes drug, is linked to cancer prevention [review in (139)]. In fact, Metformin directly inhibits mitochondrial complex I of the respiratory chain and OXPHOS activity (140). As a consequence, this drug induces cell death in cancer cells. However, this effect is observed only upon glucose deprivation. First, this study highlights the metabolic plasticity of cancer cells, which are able to survive to OXPHOS inhibition by Metformin when glucose is present. Similarly, in glutamine-addicted cells, GSCs easily switch from glutamine-based to glucose-based metabolism to sustain their survival (39). Second, to overcome their metabolic flexibilities, an efficient metabolic targeting will require the blockade of several metabolic pathways. Indeed, in preclinical cancer models, dual inhibition of both glycolysis and OXPHOS, respectively, using the glucose analog 2-DG and metformin, has been shown to effectively reduce tumor growth and dissemination (141). Similarly, phenformin, another biguanide drug, leads to cancer cell death through metabolic catastrophe only when combined with genetic disruption of MCT involved in cancer metabolic symbiosis (142).

Interestingly, since mitochondria evolved from bacteria, many classes of FDA-approved antibiotics, recently emerged as additional inhibitors of mitochondrial biogenesis and functionality (143, 144). For example, doxycycline, very well-tolerated in patients, could be re-purposed clinically as a “safe” mitochondrial inhibitor, targeting mitochondrial biogenesis in CSCs (145).

As described above, TME provides cancer cells and GSCs with various bioenergetic nutrients. At the molecular level, MCT play a key role in the metabolic symbiosis, whereby lactate produced by one cell type is made use of as a fuel by another one. Simultaneous inhibition of both MCT and OXPHOS might represent a candidate strategy for combinatorial metabolic targeting. At the cellular level, GSCs are known to closely interact with endothelial cells (66, 70). First, endothelial cells promote cancer cells stemness in GBM (146). Second, endothelial cells, and in particular radiation-resistant endothelial cells, can protect stem cells and tumor cells from radiation damage (147–149). Furthermore, we recently showed that radiation induced metabolic mitochondrial alteration in these radiation-resistant endothelial cells (150). Thus, disrupting the tumor-TME interaction that support GSCs survival is another approach to killing GSCs.

### Metabolic Targeting to Sensitize GSCs to Radiation

Hypoxic niches protect cancer cells from radiation-induced killing since oxygen is a critical determinant of cell response to radiation (151). A certain degree of reoxygenation can be achieved in some tumors after radiation by a process where the surviving hypoxic tumor cells become better oxygenated due to the aerobic population being killed. However, this process is highly variable within tumors. The HIF-1α pathway enables tumor cells to survive by changing glucose metabolism toward a glycolytic phenotype, by inducing angiogenesis and by regulating pH balance and proliferation rate.

Differentiation of normal stem cells is associated with a metabolic shift from glycolysis to mitochondrial OXPHOS while iPS reprogramming is accompanied by the reverse modifications (152, 153). Since the glycolytic switch occurs before acquisition of pluripotent markers, mitigating metabolism to induce GSCs differentiation appears as an appealing therapeutic avenue. Importantly, cancer cells, as normal cells, are more sensitive to conventional treatments, including radiation, upon differentiation (154). Differentiation therapy has been exploited with Bone Morphogenetic Protein-4 (BMP4) treatment, to induce glial differentiation and reduce tumor growth in gliomas. Interestingly, after this treatment, GSCs are unable to form tumors after transplantation in series in immunocompromised animals (155). Collectively, these studies suggest a new treatment for GBM that would force the GSCs to enter differentiation, resulting in sensitization of GSCs to treatment and as a result the reduction of the tumor mass and relapse occurrence (Figure 3). In agreement with this idea, Dichloroacetate (DCA), an indirect PDH activator, leads to both GSCs differentiation as well as increased radiation sensitivity (156, 157). One report showed that DCA, already used in the clinical treatment of genetic mitochondrial diseases, may improve clinical outcome in some patients with GBM (158). Following this study, 4 phase 1 clinical trials have investigated the chronic safety of oral DCA doses in adults with recurrent malignant brain tumors or other solid tumors (159–161). However, whereas these clinical trials showed that DCA was generally well-tolerated despite a peripheral neuropathy in some patients, there was in general no strong indication for a relevant effect of DCA in tumor response.

Other metabolic inhibitors have been involved in GSCs differentiation. As described previously, the oncometabolite D2-HG, produced by IDH mutant, is also involved in cell differentiation (162). Furthermore, inhibitors of LDH decrease GBM cell proliferation, trigger cellular apoptosis and more importantly induce GSCs differentiation (163). EGCG, a bioactive polyphenol present in green tea and described to inhibit glutamine metabolism, has been shown to decrease stemness while increasing Temozolomide efficiency (39, 164). The effect of their use in combination with radiation has not been studied yet and would be worthy of further investigation.

Other studies have shown that metabolic targeting increased treatment efficiency, independently of GSCs differentiation. For example, modulation of mitochondrial metabolism influences radiation sensitivity both *in vitro* and in preclinical models (165). The glucose analog 2-DG, which causes a significant reduction of ATP levels by inhibiting glucose catabolism especially in cells with mitochondrial defects or under hypoxia (166), has entered numerous clinical trials and seems to potentiate the effect of radiotherapy in patients with brain tumors (167). Collectively, these studies suggest that targeting metabolism may offer benefit in GBM treatment, in particular in combination with radiation. However, further investigations are definitively required in particular in preclinical studies.

### Metabolic Targeting to Improve Immunotherapy Efficacy

The immune cells being an important component of the tumor microenvironment, cancer immunotherapy has emerged as a powerful new therapeutic approach either by active, passive or adoptive immunotherapies [review in (168)] (Figure 3). Immunotherapy usually boosts antitumor immune responses either by adoptive T cells transfer, chimeric-antigen receptor T-cells, or monoclonal antibodies. For example, cytotoxic T lymphocyte-associated antigen 4 (CTLA-4) and the programmed cell death protein 1 pathway (PD-1/PD-L1) checkpoint inhibitors are currently arising as a novel strategy to fight cancer, including GBM. However, the highly immunosuppressive TME combined with low GBM cells immunogenicity limits immunotherapy efficacy. Furthermore, several recent reports have described a variety of “metabolic checkpoints” including glucose and amino acid depletion, hypoxia, high acidity and lactate accumulation, that impairs TIL ability to survive, proliferate and function (169). Interestingly, the immune checkpoint blockade favors T cell activation while inhibiting cancer cells proliferation through metabolic alterations. For example, blocking PD-1 and PD-L1 can reduce glycolysis level in cancer cells by inhibiting the mTOR pathway (170). As a consequence, tumor glucose uptake and lactate secretion decrease, restoring glucose availability in TME. Interestingly, increased glucose availability in TME in response to immune checkpoint inhibitors has been shown to improve T-cell glycolysis and cytotoxic function in a melanoma murine model (171). However, other studies have shown that the upregulation of both tumor PD-L1 and CTLA-4 drives T cells exhaustion through metabolic alterations. Recent publications also underline the reprogrammation of the immunosuppressive TME through metabolic alterations of tumor cells. The best example is the overexpression of the C4-metabolite carrier UCP2 in melanoma cells triggering the engagement of anti-tumor immune responses following CXCL10 secretion in the TME (172). Thus, tumor metabolism modulates tumor immune evasion through nutrients availability for T cells and reprogrammation of the TME.

Besides leveraging the body's own immune system, immunotherapy strategy may involve adoptive T cell transfer which offers the potential to overcome one of the significant limitations associated with tumor patients who are often immune compromised. Importantly, in such settings, Vγ2Vδ2 T cells are particularly interesting since they are able to recognize and kill most tumor cells in a major histocompatibility complex (MHC)-unrestricted fashion and independently of the number of tumor mutations. Instead, Vγ2Vδ2 T cells respond to the presence of small isoprenoid metabolites, such as self isopentenyl pyrophosphate (IPP) in a process requiring the butyrophilin-3A1 protein, an immunoglobulin superfamily protein present on all normal and tumor cells. Accumulation of IPP in tumor cells can be achieved by inhibiting the Farnesyl diphosphate synthase (FDPS), an enzyme downstream of the mevalonate pathway, by aminobiphosphonate compounds such as zoledronate. The mevalonate metabolic pathway provides cells with bioactive molecules, in particular cholesterol and coenzyme Q as well as molecules involved in signal transduction, which have been shown to be crucial for different cellular processes, including cell proliferation, differentiation, and survival [review in (110)]. In some tumor types, upregulation of several enzymes upstream of this metabolic pathway has been associated with CSCs enrichment (111). Interestingly, we have recently shown that allogeneic transfer of human Vγ9Vδ2 T cells in orthotopic murine models of human GBM enriched in GSCs leads to the efficient elimination of cancer cells, including GSCs (173). These results are in agreement with previous pre-clinical studies showing that statins could be used as anticancer agents in Glioblastoma (174, 175). Interestingly, asides from metabolic targeting, this paper also raised the idea that allogeneic transfer of human Vγ9Vδ2 could be potentiated with the combinatorial use of monoclonal GD2-antibody. Whereas, GD2 is also expressed in gliomas and in some normal structures of the brain, the monoclonal *O*-acetyl-GD2 antibody recognizes GSCs and more importantly is tumor specific (176). Thus testing the combinatorial effect of the monoclonal *O*-acetyl-GD2 antibody with adoptive transfer of human Vγ9Vδ2 should be investigated.

### The Limits of GSCs Metabolic Targeting

Several drug targeting metabolic pathways have been investigated in human randomized trials after the promising results obtained *in vitro* and in preclinical models. The majority of these metabolic inhibitors has been well-tolerated and do not interfere with normal cellular metabolism in a clinically meaningful manner. Unfortunately, few of these drugs have shown encouraging results in patients to date. *In vitro* studies provide a solid platform to include metabolism as a definite hallmark of cancers and to consider the metabolic profile of CSCs as a relevant therapeutic target. However, one difficulty is to gain integral information on which bioenergetic and biosynthetic reactions of GSCs are key players in tumor progression and/or response to therapy, in particular in light of the inter- and intra-tumor heterogeneity. Furthermore, contradictory metabolic phenotypes have been obtained between *in vitro* studies and *in vivo* studies. For example, ovarian CSCs display a strong oxidative metabolism *in vitro* while being highly glycolytic *in vivo* (177). Thus, while *in vitro* models are definitively improving to better recapitulate the TME, the lack of a relevant TME remains one of the main pitfalls in studying CSCs *in vitro*. Indeed, in this review, we provided numerous evidences of the importance of the tumor niche in driving CSCs bioenergetic and biosynthetic deregulation.

One concern about metabolic targeting is that the adaptation of metabolic pathways in conjunction with the use of alternative nutrients could overcome the targeted metabolic inhibition in GSCs. Indeed, it is likely that in many cases in which glycolysis is inhibited, cells will respond by increasing OXPHOS. One way to circumvent this compensation is to combine several metabolic inhibitors. The exploitation of the metabolic reprogramming to selectively target tumor cells may also be limited by the existence of multiple isoforms of the enzymes and the fact that small-molecule inhibitors may not distinguish between the predominant isoform expressed by cancer cells and the isoforms expressed by normal cells. Developing small-molecule inhibitors that preferentially inhibit the cancer-specific isoform could be challenging.

Metabolic targeting has minimal efficacy by itself and tumor cells may develop mechanisms to adapt and resist to metabolic inhibitors. In contrast, combination therapy seems more promising. Indeed, radiation targets highly proliferating tumor cells while sparing low-proliferating GSCs. Thus, targeting GSCs metabolism appears as new therapeutic strategies to successfully eradicate them. Some metabolic inhibitors such as inhibitors of glucose transporters, hexokinase or PKM2 agents have been clinically disappointing with no beneficial effect in GBM patients, alone or combined with radiation therapy either because of toxicity, poor tumor penetrance, or lack of efficacy *per se* (178). However, phase I/II clinical trials have shown that 2-DG administered orally was well-tolerated and triggers a moderate increase in the survival of GBM patient treated with radiation therapy, with a significant improvement in the quality of life and better protection of normal brain tissue (167). A phase III multi-centric trial to evaluate the efficacy of the combined treatment is in progress. In this regard, the use of metabolic targeting as an adjuvant therapy to increase the efficacy of conventional treatment might be a better strategy.

## Conclusion

The crucial role played by GSCs in tumor initiation, progression, recurrence, and resistance to therapy indicates that new therapeutic strategies require the eradication of this population. The inter- and intra-tumoral heterogeneities combined with the variable patterns of vascularization and nutrients availabilities might directly draw the metabolic landscape of Glioblastoma rather than oncogenic genetic events. Whereas, GSCs metabolic plasticity represents a major challenge in the design of efficient therapies, tumor metabolic targeting holds great therapeutic potential in improving cancer treatment as shown by encouraging results observed using IDH mutant inhibitors in AML. In Glioblastoma, we believe that metabolic targeting has to be used in combination with standard anticancer reagents such as radiotherapy. Thus, while a well-defined metabolic portrait of GSCs still needs to be depicted to fully exploit GSCs metabolic vulnerabilities, in particular how to prevent their metabolic flexibilities and to define the best therapeutic window combining metabolic inhibition, immunotherapy and radiotherapy, we believe that GSCs metabolic targeting holds great therapeutic potential as an adjuvant therapy.

## Author Contributions

DG and CP: manuscript writing. OR: figures drawing. M-CA-G and FP: scientific discussions and extensive proofreading.

### Conflict of Interest Statement

The authors declare that the research was conducted in the absence of any commercial or financial relationships that could be construed as a potential conflict of interest.
